# Exercise Testing Score for Myocardial Ischemia Gradation

**Published:** 2007-01-01

**Authors:** Augusto Hiroshi Uchida, Paulo Moffa, Andres Ricardo P Riera

**Affiliations:** 1 Heart Institute - University of Sao Paulo Medical School; 2 Chief of Electro-Vectorcardiographic Sector. Medicine faculty. ABC foundation. Santo Andre - Brazil

**Keywords:** exercise testing, myocardial ischemia, coronary disease

## Introduction

Scores aimed at contributing to the optimization of exercise testing (ET) have been developed and the experience with their application in coronary artery disease (CAD) has proven to be favorable [1].

Although there is debate on the use of scores in clinical practice, those that stand for it argue that they may decrease the rate of undiagnosed CAD, besides reducing the number of patients without disease that undergo highly expensive tests [2]. Additionally, scores may be helpful, in a more consistent and organized fashion, in prognosis evaluation and in the adoption of an appropriate plan of action for the triage of this disease in the general population.

Besides improving diagnostic and prognostic accuracy, scores remove interpretation biases and reduce variability in the decision-making process. Physicians frequently make clinical decisions based on their personal experience, instead of following a rational decision-making process, in addition to trusting more the results from more expensive tests, such as perfusion imaging or echocardiography. Using scores has shown great results, as good as or even better than the formerly mentioned tests [3], but the complex nature of equations intimidate physicians to make a routine use of them.

Although many scores adopt particular features of electrocardiographic responses in their composition, there are no references for a proposal to graduate myocardial ischemia documented in ET.

Most cardiologists divide the wide spectrum of electrocardiographic alterations into just two categories (normal/abnormal), which still lack a clearer definition. Such lack of a proper categorization for ischemic response, determines an inappropriate comparison of results from large studies, thus promoting interpretation biases.

To this moment, no line of research has turned primarily toward the CAD evaluation through ET, enabling a view beyond the simple dichotomy (negative/positive test), to provide more objective data about the degree of myocardial ischemia documented during the test.

In this article, we propose an electrocardiographic score for myocardial ischemia graduation during the exercise testing. This is a conceptual proposition and clinical studies must be designed to validate the new score among many populations and clinical conditions. Inter-observer variations studies and comparisons with other scoring systems and diagnostic methods will help the medical community to evaluate the reliability and better understand the clinical relevance of the new exercise testing ischemic score.

## Need for an electrocardiographic scale

Countless situations of clinical practice require graduating myocardial ischemia documented in ET. We may enumerate the following situations:

1. *Diagnostic approach to CAD*: The degree of ET positivity clearly influences the probability of coronary artery disease. The greater the magnitude of the ST segment depression, the greater the probability of CAD. Also, the worse the mophological type of ST segment depression, the more likely the presence of coronary insufficiency. The presence of ST segment elevation defines a high probability of severe CAD. Also, the earlier and the longer the ST segment depression, the higher the probability of severe CAD. Currently, patients that present ST segment depression of 1.0 mm of horizontal morphology, restricted to the maximal exercise, are mistakenly categorized in different studies, as in the same probabilistic level of disease as in individuals with ST segment elevation of great magnitude in the initial phase of exercise.
2. *Therapeutic management*: The type, the time, and the intensity of the therapeutic management vary according to the severity of the alterations documented in ET. Frequently, such classification of severity is made empirically by most cardiologists. We are missing an objective form of classification for myocardial ischemia severity for a better basis for clinical decisions.
3. *Evaluation of therapeutics*: Usually, ET are conducted to evaluate the result of different therapeutic approaches in individuals with CAD. When revascularization procedures are incomplete, graduating ischemia would be useful in monitoring clinical evolution.
4. *Risk stratification*: To better establish the risk of future events, graduating alterations in ET is essential, since there is a great range of presentations of myocardial ischemia. Certainly, an individual that presents a 1.0 mm ST segment depression with horizontal morphology restricted to maximal exercise, should not be classified in the same risk level as individuals with ST segment elevation of great magnitude in the initial phase of exercise.
  Many studies with a prognostic approach that assessed ST segment depression as an independent risk factor, presented an interpretation bias of the results, because in clinical practice, an individual that presents a positive ET, invariably ends being invasively investigated, and consequently, being submitted to revascularization procedures. Thus, the presence of myocardial ischemia during a stress test determines a therapeutic approach that modifies the natural history of coronary artery disease.
5. *Serial and comparative analysis*: A serial analysis of ET performed with the passing of time is necessary for patients who have some contraindication for myocardial revascularization. Serial monitoring of myocardial ischemia could be the basis for different adjustments of pharmacological therapeutics. A graphic demonstration of the degree of myocardial ischemia could be useful to express the patient's clinical evolution. Besides this, as clinicians are used to comparing patients with their prior condition or even patients between each other, severity gradients may have a wide range of applicability.
  Graduating myocardial ischemia may also minimize differences between results of ET and perfusion images. Early electrocardiographic alterations with great magnitude, may be even more valued than perfusion images, when there is homogeneous uptake of the radiotracer.
6. *Ischemic preconditioning (IPC)*: This is a physiological phenomenon of cardioprotection, where myocardial ischemia episodes induce a greater myocardial tolerance to a subsequent ischemic damage. This may be objectively shown by two sequential ET, where an attenuation of ischemia signs is observed in the second test [4]. IPC indicative parameters include: delay in appearance of angina symptoms and of ischemic electrocardiographic signs, less magnitude of electrocardiographic alterations, faster recovery from post-exercise alterations, improvement of hemodynamic parameters, improvement of tolerance indexes and ischemic thresholds.
  The time for 1.0 mm of ST segment depression is considered a tolerance index. Ischemic threshold indexes correspond to heart rate and the double product at the time in which 1.0 mm ST segment depression is reached.
7. *Scientific research*: To prevent great biases in the interpretation of results, an appropriate data homogenization is necessary. Graduating myocardial ischemia may reduce variability of interpretation of the results, by improving data systematization and patient classification. Scales are more systematized forms of observation, and translate a biological phenomenon in more objective and quantified information.

## Existing Electrocardiographic Scores

Scores to assess CAD may be classified didactically, as pre-test, post-test, simplified, multivariate, diagnostic or prognostic. Diagnostic scores are structured, essentially aiming at disease probability estimation. When only clinical variables are considered, this is deemed as a pre-test analysis; by including ET parameters, this is deemed a post-test score. A diagnostic score may be prognostic when severe CAD estimation is made (i,e. multi-vessel disease pattern, left truncus lesion). Prognostic scores are structured to assess risk, mainly of cardiovascular death.

There are scores based on multivariate equations that possess complex and difficult calculations. Simplified scores derived from those equations, which as a table, allow for a quick estimation of coronary artery disease through the simple addition of points. Computerized systems perform the calculations of multivariate predictive equations, which may by data weighing and logistic regression, make a diagnosis as accurate as more expensive and sophisticated tests. In spite of forecast equations being possibly intimidating for clinicians, there are programs that automatize such calculations.

Although there is a great abundance of alternatives and proposals of scores in ET, with many of them considering aspects of electrocardiographic response in their composition, a few are scores specifically aimed at electrocardiographic aspects. Within the scores that consider exclusive aspects of electrocardiographic response, those of Atenas [5] and Koide [6] are quoted. The score of Atenas is a CAD diagnostic index, based on the analysis of QRS width variations during stress test. The score of Koide has a diagnostic focus and uses 3 ET variables that should be considered in a sequential and hierarchical way: ST segment depression, QT dispersion and the result of the Atenas score.

## The Graduation Scale Proper

This is a system that depends on identifying electrocardiographic variables that may be clearly defined and graduated according to a scale of values, the points of which represent a graduation of myocardial ischemia that is documented in ET.

Within the great range of electrocardiographic alterations, this scale classifies the different patterns into 3 fundamental aspects: magnitude, morphology and moment of ST segment deviations. Each aspect is hierarchically classified into 5 types and graduated from 0 to 4 points (Table 1-3), resulting into a scale that ranges from 0 to 12 points.

## Morphological Analysis

**Figure 1:**
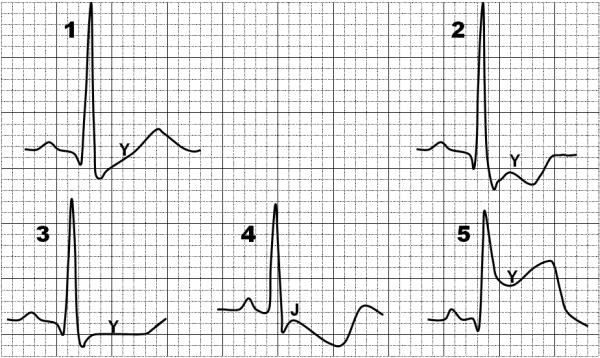
Morphological patterns of ST segment deviations considered for ischemia score composition.

The electrocardiographic interpretation should be systematic and comprehensive, weighing globally the findings for a proper correlation establishment with morphological types defined by the scale of ischemia. There are 4 morphological patterns of depression and one morphological type of ST segment elevation (Figure 1):

1. *Upsloping ST segment depression*: The rapid and slow upsloping patterns are classified within this item. The point of reference for measuring ST segment depression in the upsloping type is the Y point, at 80 ms from the J point (J80).
2. *ST segment convex depression*: The presence of ST segment convexity characterizes this morphological pattern. It should be measured at the Y point, at 80 ms from the J point (J80).
3. *ST segment horizontal depression*: It should be measured at the Y point, at 80 ms from the J point (J80).
4. *ST segment downward depression*: It should be measured at the J point.
5. *ST segment elevation*: It should be measured at the Y point, at 40 ms from the J point (J40).

## Definition of ST Segment Deviations Magnitude

The measurement should be conducted according to a morphological pattern of ST segment deviations. The lead with the greatest alteration is adopted for scoring. To make up the score, the magnitude of deviations is classified into 5 categories:

1. *No ST segment deviations*. The point of reference (J or Y) is in the PQ baseline or keeps the same magnitude of the baseline situation.
2. *ST segment shift of small magnitude*. ST segment depression or elevation inferior to 1.0 mm.
3. *ST segment shift from 1 to 1.5 mm*. ST segment depression or elevation between 1.0 mm and 1.5 mm.
4. *ST segment shift from 1.6 to 2.0 mm*. ST segment elevation or depression superior to 1.5 mm and inferior or equal to 2.0 mm.
5. *ST segment shift superior to 2.0 mm*. ST segment depression or elevation of great magnitude.

## Moment and Duration of Alterations

**Figure 2:**
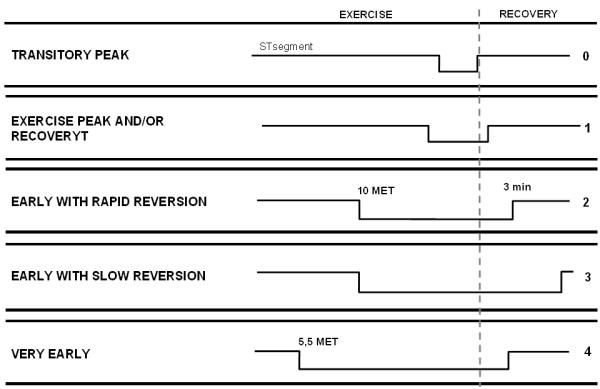
Patterns related to the time and duration of ST segment deviations for the analysis of ischemia score.

To score this component, 5 patterns are considered that are exemplified in Figure 2:

1. *Transitory peak*. When ST segment shift occurs exclusively in the phase of exercise, appearing after 10 MET, which corresponds to stress test that is altered after the third stage of Bruce's protocol. The total resolution of ST segment elevation should occur before the first minute of recovery.
2. *Peak and/or recovery*. It corresponds to tests where ST segment deviations appear after 10 MET or after the third stage of Bruce's protocol, with a total resolution of ST segment shift after the first minute of recovery. ST segment deviations that occurred exclusively in recovery should also be considered in this pattern.
3. *Early with rapid reversion*. When ST segment shift occurs between 5 and 10 MET, with total resolution of ST segment shift that occurs before the third minute of recovery.
4. *Early with slow reversion*. When ST segment shift occurs between 5 and 10 MET, with total resolution of ST segment shift that occurs after the third minute of recovery.
5. *Very early*. When ST segment shift occurs with up to 5 MET, corresponding to alteration that occurs in the first stage of Bruce's protocol.

## Methodological Reflections on Analysis

### Electrocardiographic tracing quality

To validate ST segment alterations that characterize myocardial ischemia, the following should be used: morphological data, referential baseline stability, tracing quality, definition and number of leads involved, type and calibration of the equipment and recording system.

ET are considered to be satisfactory when they present recordings in a representative amount, such that visualization would enable a diagnostic conclusion. ET are considered to be unsatisfactory when their reading is hampered by the presence of interference, artifacts or wide variations of the baseline. To validate interpretation, there should be at least three beats with a stable baseline. Electrocardiographic alterations determined by breathing alterations, movements and variations of the baseline, all should be excluded of the morphological analysis.

### Recording system

Using only 3 simultaneous leads substantially hinders the sensitivity to detect myocardial ischemia, and using bipolar precordial leads increases sensitivity but decreases specificity due to a greater amplification of the electrocardiographic signal. Inappropriate equipment may produce artifact alterations, magnify or mask alterations by problems of acquisition, processing or even inappropriate graphic printing of electrocardiograms.

Leads involved in morphological alterations. Specific leads and the number of leads should be pondered in global interpretation. When morphological alterations affect isolated leads, without comprising a region; or affect a not so relevant lead (e.g. aVR), such alterations should not be validated to define ischemia. Leads with a simultaneous recording should be compared to assess the equivalence of alterations.

### Reference points

The measurement of any ST segment shift is made from the PQ baseline and not from the PR line. ST segment depressions with downsloping morphology are measured at the J point, and horizontal, upsloping and convex patterns are measured at the J80 point. ST segment elevations should be measured at J40 point.

### ST segment depression

The slow upsloping pattern is considered abnormal; however, it was excluded as positivity criterion, since it determines a high rate of false positives. The convex pattern is not so acknowledged in international literature, and it determines a high rate of false positives as well; it normalizes rapidly in recovery, before the first minute. Classically, the horizontal and downsloping patterns are the ones that define the internationally accepted positivity criteria, and the downsloping pattern indicates a more severe ischemia [7].

It is worthwhile to highlight that positivity criteria are the same for both genders, since the electrocardiographic manifestation of ischemia is not different between men and women. What changes is the CAD probability. The probabilistic analysis of coronary disease involves not only gender, but also age, symptoms and other numberless risk factors. These numberless variables may be organized and classified through scores, indexes or diagnostic and/or prognostic scales.

## Specific Situations

### Baseline ECG alterations

ECG morphological alterations in rest should be weighed in interpreting electrocardiographic response. In the case of baseline ST depression, ST depression line is adopted as a reference to measure the possible additional shift during exercise. In the cases where baseline ECG presents great morphological alterations, with depressions greater than 1.0 mm, there is limitation of morphological analysis to define myocardial ischemia. Morphological interpretation of ECG to define ischemia should also be considered limited in the following situations: use of digitalis, left ventricular enlargement, intraventricular conduction disorder, ventricular pre-excitation and long QT.

### Q wave

Q wave increases physiologically during exercise. When there is doubtful ST segment depression, observing Q wave reduction should be applied to validate the alteration.

The presence of pathological Q wave in baseline ECG should be considered to interpret the possible ST segment elevation in stress test. Once ST segment elevation occurs, with magnitude criterion, in the presence of electrically inactive area, dyskinesia should be investigated. To make up the score for ischemia, ST segment elevations with areas with pathological Q waves should not be considered.

### Early repolarization

In the case of early repolarization pattern in baseline ECG, the measurement of possible ST segment depression is considered from PQ baseline.

### Baseline ST segment elevation

When baseline ECG presents ST segment elevation in electrically inactive area, the reference line for measurement is considered to be the level of elevation to interpret possible additional elevations. As a general rule, ST segment elevations are measured at 0.40 sec from the J point.

### Right bundle branch block (RBBB)

In the presence of baseline RBBB, the presence of ST segment depressions is only validated in the V5 and V6, D1, aVL, D2, D3 and aVF leads.

### Left bundle branch block (LBBB)

LBBB hinders morphological analysis to define myocardial ischemia. Morphometric analysis of ST segment deviations (elevation or depression) does not allow characterizing myocardial ischemia in most cases.

### Memory effect in recovery

ST segment morphological alterations that occur after transitory episodes of branch blocks or tachyarrhythmias, are considered to be a memory effect (electronic memory), and are not considered to define myocardial ischemia.

### Post-extrasystolic beats

Altered beats that follow early ventricular or supraventricular beats, are not considered for morphological analysis.

### Nonspecific alterations

Alterations that may be correlated to myocardial ischemia in other contexts. E.g.: T wave pseudo-normalization and inversion; they are not considered in the composition of ischemia score. Of note, late inversions of T wave in the recovery phase are frequently mistaken by ST segment depressions with downward morphology.

### QRS magnitude

QRS complex of great magnitude (very broad R wave), which determines greater magnitudes of ST segment deviations. A small ST alteration may be magnified when the R wave is large. When there is low QRS voltage, with R wave amplitude inferior to 11 mm, the rate of false negatives is greater [8]. Thus, small ST alterations should be more valued.

### Left ventricular enlargement (LVE)

Hypertensive patients, with baseline ECG alterations compatible to LVE, have a positive predictive value of impaired test. Anyway, a normal ST in LVE carriers has a high negative predictive value for coronary artery disease. While there is LVE, ST segment alterations should not be considered for the ischemia score.

### Influence of  Medication

Digitalis and other antiarrhythmic drugs substantially affect ventricular repolarization, limiting morphological analysis to define myocardial ischemia and invalidating the use of the score for ischemia. It is essential to weigh the result of the test when these specific medications are used, or even when there are still residues of therapeutic influence.

### Sustained tachyarrhythmias

After episodes of tachyarrhythmias, whether supraventricular or ventricular, ventricular repolarization alterations may be observed that resemble myocardial ischemia. Thus, morphological alterations that occur immediately after sustained tachyarrhythmia episodes are not considered for ischemia score composition.

### Bradyarrhythmias

Atrioventricular and sinoatrial blocks are not directly correlated to myocardial ischemia, even when transitory during exercise. They should not be considered for ischemia score composition.

### Visual vs. computerized method

The analysis of electrocardiographic response should be made by the visual method instead of the computerized method, which is not considered to validate the interpretation due to the high degree of contamination by artifacts.

### Post-exercise or recovery phase

Prolonged cool down (period of active recovery, walking slowly) may obscure possible early ST segment alterations.

### Atrial T wave (Ta)

Accelerated atrioventricular conduction favor Ta wave expression, which manifests as ST segment depression with a pattern similar to the slow rising one. Such depression is more emphasized in D2, D3 and aVF, and should be considered as an electrocardiographic response deviant from normal [9].

## Discussion

As the morphological interpretation of the electrocardiogram is subjective, and there is a large amount of data that are manipulated during the process of analysis, there has been an increasing need to systematize and organize the process of data interpretation.

Over the last decades, cardiologists are focused on expensive and sophisticated diagnostic methods, in the belief that they can provide a better diagnostic and/or prognostic accuracy. Collecting scientific data, it is still possible to verify that conventional ET can, most of the times, be superior to more recent tests when scores are used [3].

There are many bases or scores to classify CAD based on the duration of signs or symptoms or in anatomical location and in the extension of the atherosclerotic plaque [10]. Also in the areas of myocardial perfusion images [11], in echocardiography [12] and in magnetic resonance [13] perfusion scores are used as a routine. In electrocardiography, a graduation system for myocardial ischemia was previously proposed, to be applied in the setting of acute myocardial infarction [14,15]. Anyway, there is no similar system to be applied in the field of ET.

A score, besides systematizing and organizing the interpretation of the data from the ET, may be a useful quantifying instrument for alterations. The proposed scale for ischemia may be understood as an optimized score, where there are legitimized entries of data classified by applications in different specific populations.

Scales are useful to characterize and quantify, i.e. translate better the clinical phenomenon into objective and quantified information. The graduation system for ischemia that we propose, categorizes the response in patterns based on three predominant aspects that, when added, result in a scale or score. This system enables a description of the extension of myocardial ischemia, and even in a carrier of defined CAD, may involve a given number of correlative objectives, namely: help the physician to plan the treatment; provide a better prognostic definition; help to assess the results of the treatment; facilitate the exchange of information between the treatment centers; contribute with the continuing research about myocardial ischemia.

It is important to reach a greater harmony in the record of data that result from ET, and promote a more precise frame for the extension of ischemia, to correlate it better with the most diverse patterns of coronary anatomy involvement, facilitating the exchange of information between different centers as well.

Scientific research is the classical territory to use scales, thus guaranteeing that the information collected about specific alterations is organized in patterns and feasible of comparison in a reliable, consistent and reproducible fashion.

The graduation system proposed primarily works with classification of myocardial ischemia documented by electrocardiogram during ET. The score has three components: magnitude, morphology and moment of alterations. All these aspects are easily evaluated, thus allowing a good integration of information between clinicians and specialists. Although the different parameters of this scale are already evaluated as a routine, and were previously studied [16], there is no description of the analysis of the electrocardiographic response in the formal and organized way we are proposing.

This system will enable structuring more objective and optimized criteria, permitting a better discrimination and classification of patterns of the different electrocardiographic expressions of myocardial ischemia, one of the most representative paradigms of the multifaceted disease.

Myocardial ischemia is an expression of CAD and it affects the electric activity of the heart, which represents the documentary proof of ischemic disease. To be able to document myocardial ischemia, there is a series of factors that should be considered: presence of critical coronary obstruction, number of coronary arteries with critical lesions, presence of collateral circulation, location of coronary obstruction, extension of ischemia, insufficient increase of myocardial demand of oxygen, anemia, concomitant valve diseases, superimposed coronary spasm, hydroelectrolytic disorders, number of electrocardiographic leads, position of electrodes, limitations in acquisition and processing of cardiac electric signal, baseline electrocardiogram alterations, quality of electrocardiographic recording, QRS complex width, therapeutic influence, etc.

The result of the ST does not confirm the presence or absence of CAD, and it should be correlated with other pertinent data for a more coherent probabilistic analysis [17]. As any diagnostic method, ET have limitations, displaying both false-positive and false-negative errors.

Tests considered negative are not a synonym of normal tests, since they include true negatives and false negatives. Besides this, even true negatives include situations of normal coronary arteries and coronary arteries with non-critical lesions. Therefore, positive tests are not synonyms of abnormal tests, since they contemplate true-positive and false-positive results. And, even true positives include situations of critical coronary artery disease and angiographically normal coronary arteries (microcirculatory disease).

For a system to be widely accepted, it needs to be practical, easy to remember and interpret, both by the clinician and the specialist. Anyway, seeking simplicity cannot determine analysis errors and regard alterations in an absolute way. The score for ischemia we propose, focuses on the essential aspects of the ET results, with a representative and consistent character, and in an easily understandable fashion. Besides this, even physiological manifestations are considered: when the score is zero, it means that there was no documentation of myocardial ischemia.

The scale does not include angina pectoris that manifests during the test, because we consider that symptoms are described in a very subjective way for qualification and quantification, with wide inter-observer interpretation variations. Other clinical factors were not considered, because clinical information is not commonly available, or it is incomplete or uncertain.

As concomitance of highly severe hemodynamic alterations is very frequent with more severe ST segment deviations, indexes such as inotropic deficit and chronotropic incompetence were not considered for the score.

We do not oppose detailed analysis of clinical, hemodynamic and functional capacity variables, and integrating data as a whole. We just think that the magnitude of electrocardiographically documented myocardial ischemia surmounts other clinical and hemodynamic parameters besides displaying a greater reproducibility as well.

Critical aspects to use the score include:

The scale cannot be applied in situations where there are limitations of morphological analysis to define myocardial ischemia;
It is necessary for the cardiologist to be initially trained for a routine use of this score of ischemia, taking into consideration the homogenization of possible conceptual differences;
There may be small imprecision in measuring the magnitude of ST segment deviations;
As the morphological interpretation of the electrocardiogram is subjective, some ventricular repolarization patterns, even deviations from normal, may be confused in the score of morphological aspects. Less objective items tend to yield differences in scores, which may result in inter- and intra-observer variations.

In fact, it is to be expected that there should be imperfections in the proposed system, but this is a proposal that better clarifies and classifies the different aspects of myocardial ischemia. With a routine use of ischemia score, different observers may elicit the different aspects of the scale with a high degree of consistency, determining a low probability of inappropriate categorization, and reducing inter-observer discrepancies. Training on the application of scales in clinical assays, may favor a better systematization of data.

In practice, this ischemia score may prevent badly structured and disorganized clinical cases from resulting in ambiguities and misunderstandings, positioning the patient in a better defined prognostic and/or diagnostic category.

## Figures and Tables

**Table 1:**
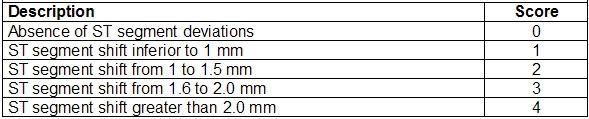
Magnitude of ST segment deviations

**Table 2:**
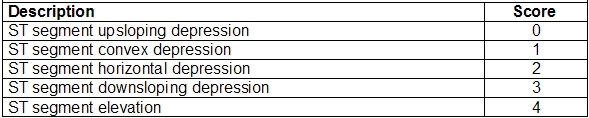
Morphology of ST segment deviations

**Table 3:**
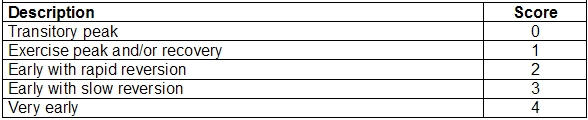
Moment of ST segment deviations
